# Insufficient tuberculosis treatment leads to earlier and higher mortality in individuals co-infected with HIV in southern China: a cohort study

**DOI:** 10.1186/s12879-020-05527-0

**Published:** 2020-11-23

**Authors:** Zhigang Zheng, Eric J. Nehl, Chongxing Zhou, Jianjun Li, Zhouhua Xie, Zijun Zhou, Hao Liang

**Affiliations:** 1 Department of Epidemiology and Statistic, School of Public Health, Guangxi Medical University, Nanning, 530021 Guangxi China; 2 AIDS Program, Guangxi Zhuang Autonomous Region Center for Disease Control and Prevention, 18 Jin Zhou Road, Nanning, 530028 China; 3 Department of Behavioral Sciences and Health Education, Rollins School of Public Health, Emory University, Atlanta, 30322 GA U.S.A.; 4 HIV/TB Treatment Department, the Fourth Hospital of Nanning City, Nanning, 530023 China; 5 Guangxi Key Laboratory of AIDS Prevention and Treatment, School of Public Health, Guangxi Medical University, Nanning, 530021 Guangxi China; 6 Guangxi Collaborative Innovation Center for Biomedicine, Life Sciences Institute, Guangxi Medical University, No.22, Shuangyong Road, Qingxiu District, Nanning, 530021 Guangxi China

## Abstract

**Background:**

Tuberculosis (TB) and Acquired Immune Deficiency Syndrome (AIDS) are leading causes of death globally. However, little is known about the long-term mortality risk and the timeline of death in those co-infected with human immunodeficiency virus (HIV) and *Mycobacterium tuberculosis* (MTB). This study sought to understand the long-term mortality risk, factors, and the timeline of death in those with HIV-*Mycobacterium tuberculosis* (MTB) coinfection, particularly in those with insufficient TB treatment.

**Methods:**

TB-cause specific deaths were classified using a modified ‘Coding of Cause of Death in HIV’ protocol. A longitudinal cross-registration-system checking approach was used to confirm HIV/MTB co-infection between two observational cohorts. Mortality from the end of TB treatment (6 months) to post-treatment year (PTY) 5 (60 months) was investigated by different TB treatment outcomes. General linear models were used to estimate the mean mortality at each time-point and change between time-points. Cox’s proportional hazard regressions measured the mortality hazard risk (HR) at each time-point. The Mantel-Haenszel stratification was used to identify mortality risk factors. Mortality density was calculated by person year of follow-up.

**Results:**

At the end point, mortality among patients with HIV/MTB coinfection was 34.7%. From the end of TB treatment to PTY5, mortality and loss of person years among individuals with TB treatment failure, missing, and adverse events (TBFMA) were significantly higher than those who had TB cure (TBC) and TB complete regimen (TBCR). Compared to individuals with TBC and with TBCR, individuals with TBFMA tended to die earlier and their mortality was significantly higher (HR_TBFMA-TBC_ = 3.0, 95% confidence interval: 2.5–3.6, HR_TBFMA-TBCR_ = 2.9, 95% CI: 2.5–3.4, *P* < 0.0001). Those who were naïve to antiretroviral therapy, were farmers, had lower CD4 counts (≤200 cells/μL) and were ≥ 50 years of age were at the highest risk of mortality. Mortality risk for participants with TBFMA was significantly higher across all stratifications except those with a CD4 count of ≤200 cells/μL.

**Conclusions:**

Earlier and long-term mortality among those with HIV/MTB co-infection is a significant problem when TB treatment fails or is inadequate.

**Supplementary information:**

**Supplementary information** accompanies this paper at 10.1186/s12879-020-05527-0.

## Background

Tuberculosis (TB) and Acquired Immune Deficiency Syndrome (AIDS) are leading causes of death globally. The World Health Organization (WHO) estimates that there were 10.4 million new cases of TB in 2016 and 10% of these people were co-infected with the human immunodeficiency virus (HIV). On its own, in the same year, it was estimated that there were 1.3 million TB deaths among HIV-negative people, with an additional 374,000 TB deaths among those HIV-positive. Additionally, among the 36.7 million people estimated to have been living with HIV worldwide [1], and 1 million who died from HIV-related causes, a full 40% were caused by TB in 2016 [2].

HIV infection increases the progression of latent TB infection to active TB disease [3] and accelerates TB disease [4]. *Mycobacterium tuberculosis* (MTB) infection increases the risk of progression from HIV to AIDS and death in HIV patients [5, 6]. Improving treatment outcomes for antiretroviral therapy (ART) and anti-tubercular therapy are crucial for decreasing TB-related mortality among those with HIV/MTB co-infection. In 2010, WHO updated ART guidelines for HIV infection adults and adolescents, recommending that ART for all HIV-infected individuals with active TB, irrespective of their CD4 + T cell (CD4) count and that TB treatment should start first, followed by ART as quickly as possible afterward [7]. Following these guidelines, ART coverage improved rapidly worldwide; with 85% now on ART [1]. However, despite recommending ART and anti-tubercular therapy among patients presenting with TB and HIV, mortality associated with both diseases remains substantial [8] and the goal of halving TB-related deaths from 1990 levels by 2015 in high HIV/MTB co-infection prevalence countries has not been met.

China ranks in the top two of 22 high HIV/TB burden countries [1], and the overall prevalence of TB infection among those HIV positive is estimated to be 7.2% (range: 4.2–12.3%) and is even higher (22.8%) among AIDS patients [9]. Guangxi, a province of Southern China, bears the highest HIV/MTB burden in China, with 11% TB prevalence among those with HIV and 3.3% HIV prevalence among TB patients [9]. Furthermore, results of other research have shown that TB prevalence among those with HIV/AIDS was 17.7% [10]. Although the latest treatment outcome data showed TB treatment success rates of 84% and ART initiation of 88% for those with HIV/TB coinfection in a 2015 cohort in China [1], the challenge of high mortality among those with HIV/MTB co-infection remains, with recent studies showing that most AIDS-related deaths were due to TB infection [10]. Specifically, over 19% of AIDS-related deaths of those who were diagnosed and then died in the same calendar year in Guangxi have been attributed to TB [11]. However, little is known of the effects of TB treatment outcomes on long-term mortality of HIV/MTB co-infection globally. Insufficient TB treatment likely relates to ineffective TB infection control which leads to increases in mortality. But, the factors related to the high mortality among those with HIV/MTB coinfection who have initiated ART and anti-TB therapy have not been well described. Therefore, the goal of this study was to evaluate the impact of TB treatment outcomes on the long-term mortality of HIV/MTB co-infection, particularly in those who are with insufficient TB treatment.

## Materials and methods

### Study setting

This study was carried out in Guangxi, Southern China. The number of HIV/MTB co-infection in this province constitutes more than 30% of the cases in China. Following international guidelines, in 2010 coordinated registration and care systems were initiated between the previously uncoordinated HIV and TB programs. Specifically, following WHO policies, those diagnosed with TB are tested for HIV and those initially diagnosed with HIV/AIDS are screened for TB annually in this province. Additionally, care services in Guangxi such as ART, anti-tubercular treatments, isoniazid preventive therapy, and infection control are now coordinated with the Chinese national HIV and TB programs.

### Study design and participants

Patient data were considered for this study if they were 18 years or older and diagnosed with both HIV and MTB (including pulmonary TB and extra pulmonary TB). Their follow-up records were reviewed every 6 months to confirmed mortality in either HIV/AIDS or TB registration systems. Records were excluded when HIV or TB status was missing across registration systems and when patients listed their address to be overseas, Hong Kong, Macao, or Taiwan.

### Patient diagnosis and data collection

Sequential screening strategies were used to prevent false positives for both MTB and HIV. For MTB, clinic doctors first took a chest X-ray (CXR), then three sputum samples or chest washing fluid samples were collected for smearing, Ziehl-Neelsen staining, and then microscopy was used to screen for MTB; samples were cultured for MTB if the smear was negative. MTB infection was diagnosed if acid-fast staining positive or positive isolation of culture was detected. For HIV, blood samples were collected and ELISA tests were used to test for HIV antibodies, followed by the western blot method to confirm HIV infection.

Data collection: 14,293 unique patients were diagnosed and registered as being HIV/AIDS positive in 2011. We launched a cross-registration-systems check across the HIV treatment database, follow-up database, and registration database for MTB/HIV co-infection. Patients were classified as being co-infected with MTB/HIV if their TB infection status was confirmed in any of the three databases. We imputed the total MTB/HIV co-infection cases from HIV databases to the TB registration database, which included 42,205 TB cases, and obtained 2351 co-infection cases for analysis.

The 60-month (PTY5) follow-up period was calculated from the date of patient registration; for patients who died or were missing, the date of death or the date the patient was reported missing was considered the end-point of the follow-up. Standard follow-up information was recorded by the program staff and reported via an Internet-based system, including patient ID, time of follow-up, CD4 count, WHO clinical disease stage, and adverse events. After the recommended treatment duration (6 months for cases of drugs-susceptible TB, and one and a half year or longer for drug resistant and extra pulmonary TB) treatment outcomes were evaluated using sputum smear microscopy, CXR, and assessment of clinical symptoms. Patients were classified for this study according to these TB treatment outcomes including: TB cure (TBC) if they had completely recovered, TB complete regimen (TBCR) if they took their pills for the duration of the treatment period but did not recover completely, and TB treatment failure, missing, and adverse events (TBFMA) if they did not exhibit any improvement with their smear, had missing data, or reported experiencing adverse events. If a patient was lost to follow-up (3 months’ absence from a clinic since last visit), a site visit of the participant’s address was conducted by clinic staff to confirm their absence or death. Information collected at the site visits included the date of the last visit to the clinic or date of death.

### Statistical analyses

Data Source: Data were stratified for analysis based upon literature related to the topic and preliminary bivariate analysis. Age, gender, ART status, CD4 count, occupation, ethnicity, and route of infection were used as stratification factors. To identify potential confounding factors, bivariate Mantel-Haenszel hierarchical analysis was used to calculate the odds risk (OR) for each stratification factor. Mortality was found to be not statistically different between TBC and TBCR groups, so TBC and TBCR groups were combined to compare to those with TBFMA. We then compared the OR values for stratification variables between TBFMA and TBC/TBCR group to identify confounding factors. Confounding factors were confirmed if the difference in OR values was found to be statistically significant between TBFMA and the TBC/TBCR groups.

Analysis indices: A general linear model for mortality measurements was used for each clinical time-point [6 months (end of TB treatment), 12 months (PTY1), 24 months (PTY2), 36 months (PTY3), 48 months (PTY4), and 60 months (PTY5)] to generate restricted maximum likelihood estimates. Mortality and mortality hazard risk were compared over the 60 month period using Cox regression models; a descriptive epidemiologic method was used to describe the characteristics; a chi-square test was used to compare the differences in demographic characteristics of TBC, TBRC, and TBFMA; and a hierarchical analysis method was used to identify confounding factors, as described above. Adjusted HR values were calculated by removing those who died within the initial 6 months of therapy; adjusted OR values were calculated across the selected subgroups (adjusted for age, gender, ART, CD4 count).

Data were analyzed with R (version 3.2.2, R Foundation for statistical Computing, Vienna, Austria) and SPSS 22.0, the significance level was set at 0.05, and all hypothesis tests were two-sided.

## Results

### Study subject characteristics

**Fig. 1:**
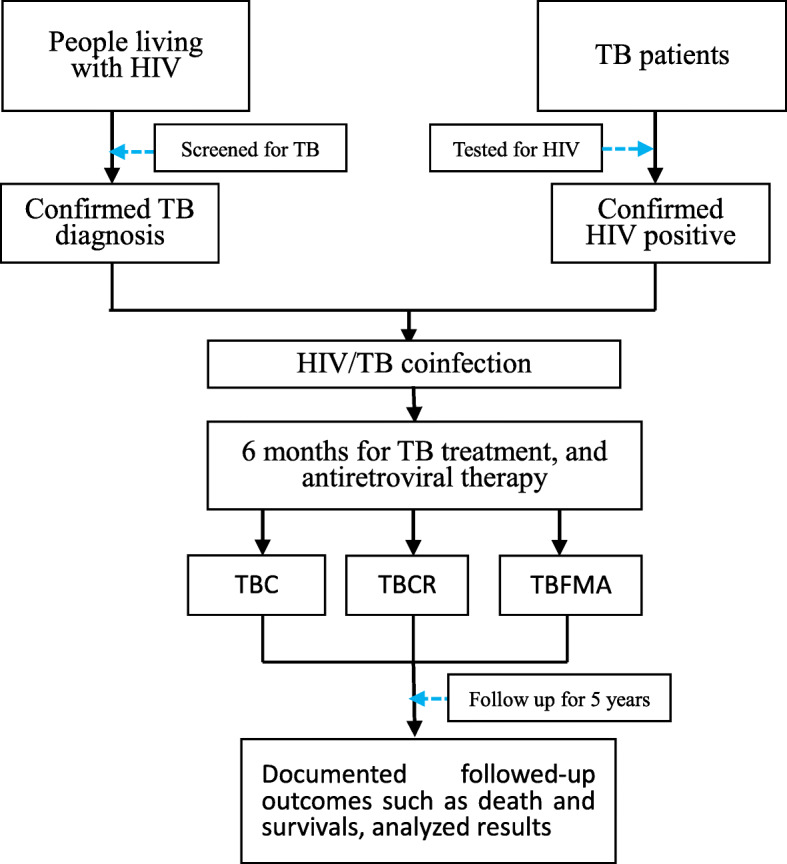
Study profile diagram showed how the study targets were selected and followed-up

Two thousand three fifty-one MTB/HIV co-infections were confirmed among 42,205 TB registered cases in 2011, and thus HIV prevalence among those with TB was 5.6% (2351/42,205); a total of 2579.7 years was provided for the TBC group at the follow-up assessment, 4872.8 years for the TBCR group, and 913.5 years for the TBFMA group over the 60-month follow-up period (Fig. 1). The median (M) time participants were tracked at follow-up for the TBC group was 5.0 person-years (PYs), and the inter-quartile range (IQR) was 2.2–5.0; the M (IQR) for TBCR and TBFMA groups was 5.0 PYs (2.1–5.0) and 1.1 (0.3–5.0), respectively (Supplemental Fig. 1). The male-to-female ratio among participants was 1.88:1; those who reported that they were farmers (75.1%) and were of Han ethnicity (58.2%) were in the majority.

### Mortality trend, hazard risk of mortality

In the TBC, TBCR, and TCFMA groups, the CD4 count (IQR) was 223 cells/μL (53.0–417.0), 184 cells/μL (56.0–367.0), and 105 cells/μL (25.50–268.50), respectively. The percentage of patients by group on ART were 53.2, 53.5, and 33.3%; and the M (IQR) of age was 45.0 (31.0–58.0), 48.0 (32.5–62.0), and 53.0 (9.0–67.0), respectively. Finally, differences in the distributions of age, gender, ART initiation, and CD4 count were statistically significant among TBC, TBCR, and TCFMA groups (Table 1).

**Table 1:** Demographic characteristics of HIV/MTB co-infection patients by treatment outcomes in Guangxi, Southern China

Characteristics	TB cure n (%)	TB complete treatment n (%)	TB treatment failure, patient missing, adverse events n (%)	*P* value
All patients	680 (28.92)	1289 (54.83)	382 (16.25)	
Age (year)				
Median	45.00	48.00	53.00	< 0.0001
IRQ	31.00–58.00	32.50–62.00	39.00–67.00	
Gender				< 0.0001
Male	455 (66.91)	796 (61.75)	284 (74.35)	
Female	225 (33.09)	493 (38.25)	98 (25.65)	
Ethnicity				0.30
Han	403 (59.26)	734 (56.95)	231 (60.47)	
Zhuang	253 (37.22)	523 (40.57)	136 (35.60)	
Yao	12 (1.76)	21 (1.63)	10 (2.62)	
Others	12 (1.76)	11 (0.85)	5 (1.31)	
Occupation				< 0.0001
Farmer	538 (79.12)	929 (72.07)	306 (80.10)	
Government employee	19 (2.79)	91 (7.05)	16 (4.19)	
Worker	15 (2.21)	38 (2.95)	9 (2.36)	
House nursing	44 (6.47)	74 (5.74)	29 (7.59)	
Farmer worker	21 (3.09)	25 (1.94)	4 (1.05)	
Student	12 (1.76)	31 (2.40)	0 (0.00)	
Others	31 (4.56)	101 (7.85)	18 (4.71)	
Infection route				0.73
Heterosexual	547 (80.44)	1004 (77.89)	282 (73.82)	
Homosexual	11 (1.62)	22 (1.71)	3 (0.79)	
IDU	35 (5.15)	61 (4.73)	14 (3.66)	
Blood transfusion	13 (1.91)	17 (1.32)	3 (0.79)	
Others	74 (10.88)	185 (134.35)	80 (20.94)	
ART				< 0.0001
Initiated ART	362 (53.24)	689 (53.45)	127 (33.25)	
ART naïve	255 (37.50)	450 (34.91)	182 (47.64)	
Unknown	63 (9.26)	150 (11.64)	73 (19.11)	
CD4 Count				
Median	223	184	105	0.002
IRQ	53.0–417.0	56.0–367.0	25.50–268.50	

Abbreviation: *IRQ* Interquartile Range; *IDU* Injection drugs users; *ART* Antiretroviral therapy

**Fig. 2:**
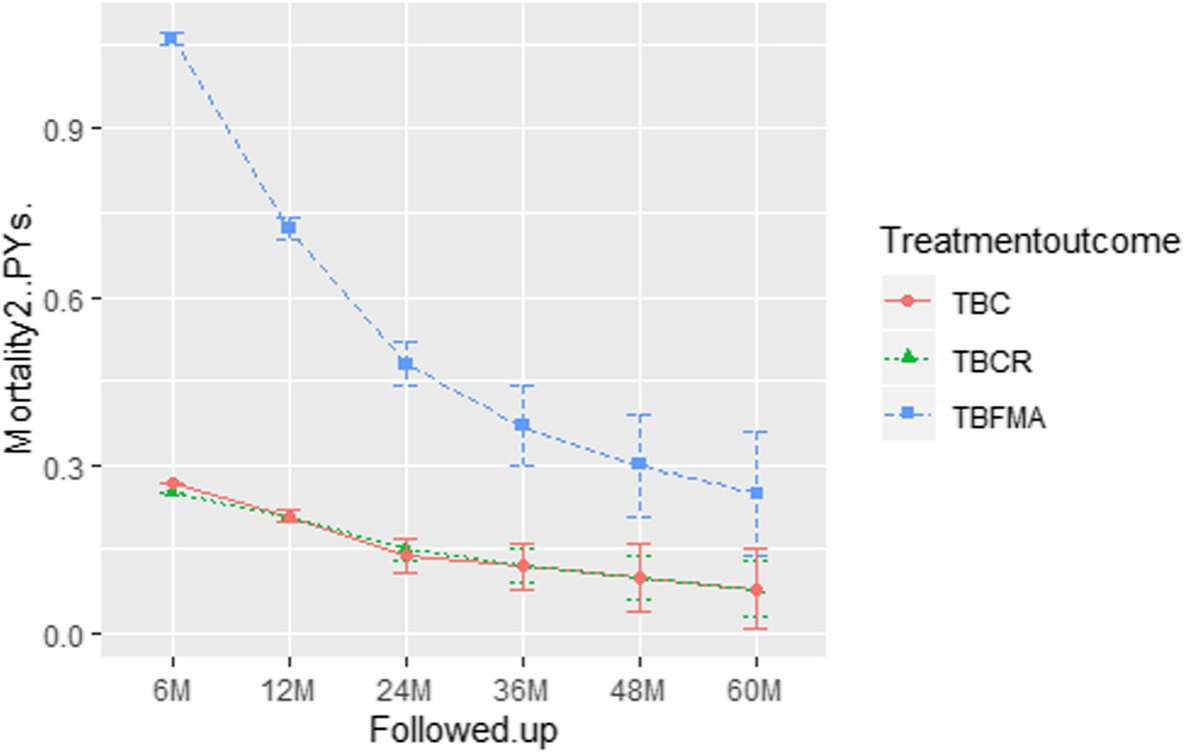
Mean mortality with ±95% confidence interval (CI) for person years of followed-up at the end of TB treatment (6 M), post-treatment year 1 (12 M), post-treatment year 2 (24 M), post-treatment year 3 (36 M), post-treatment year 4 (48 M), and post-treatment year 5 (60 M) among HIV/MTB coinfection patients treated with tuberculosis cure (TBC) (*n* = 680), tuberculosis complete regimen (TBCR) (*n* = 1289), and tuberculosis treatment failure, patients missing, adverse events (TBFMA) (*n* = 382) in Southern China

At the end point of the 60-month follow-up, a total of 859 HIV/MTB co-infected individuals had died, for a mortality of 36.5%; crude mortality in TBFMA, TBRC, and TBC groups was 60.0% (229/382), 32.0% (413/1289), and 31.9% (217/680). Compared to the TBC group (*χ*^*2*^ = 78.9, *P* < 0.0001) and TBCR group (*χ*^*2*^ = 97.0, *P* < 0.0001), the crude mortality in the TBFMA group was significantly higher (see Table 2). In the initial 6 months of anti-tubercular treatment, mortality in the TBFMA group was 41.1% (157/382), while mortality in TBC and TBCR groups was 12.5% (85/680) and 11.6% (150/1289). There was no statistically significant difference between mortality in TBC and TBCR groups, whether at the 6-month or 60-month observation (*χ*^*2*^_*6m*_ = 0.60, *P* = 0.44; *χ*^*2*^_*60m*_ = 0.003, *P* = 0.95) (Table 2). However, the initial 6-month mortality in the TBFMA group was significantly higher than in TBC and TBCR groups (*χ*^*2*^ = 118.9, *P* < 0.0001). As mortality was calculated by PYs, a declining trend was observed among all groups from 6-month to 60-month, mortality in those with TBFMA was significant higher than it was with TBC and TBCR at all time-points (Fig. 2) (Supplemental Fig. 2).

**Table 2:** Crude mortality of HIV/MTB co-infection across 60 months by treatment outcomes

Treatment outcomes	Case number (%)	Mortality in 6 months (%)	Mortality in 12 months (%)	Mortality in 24 months (%)	Mortality in 36 months (%)	Mortality in 48 months (%)	Mortality in 60 months (%)	χ^2^	*P* value
Adverse events, failure, missing	382 (16.25)	157 (41.10)	187 (48.95)	212 (55.50)	222 (58.12)	227 (59.42)	229 (59.95)	107.8	< 0.0001
Complete treatment	1289 (54.83)	150 (11.64)	242 (18.77)	315 (24.44)	379 (29.4)	404 (31.34)	413 (32.04)	–	–
Cure	680 (28.92)	85 (12.50)	126 (18.53)	164 (24.12)	192 (28.24)	206 (30.29)	217 (31.91)	–	–

Abbreviation: *IRQ* Interquartile Range; *IDU* Injection drugs users; *ART* Antiretroviral therapy

**Fig. 3:**
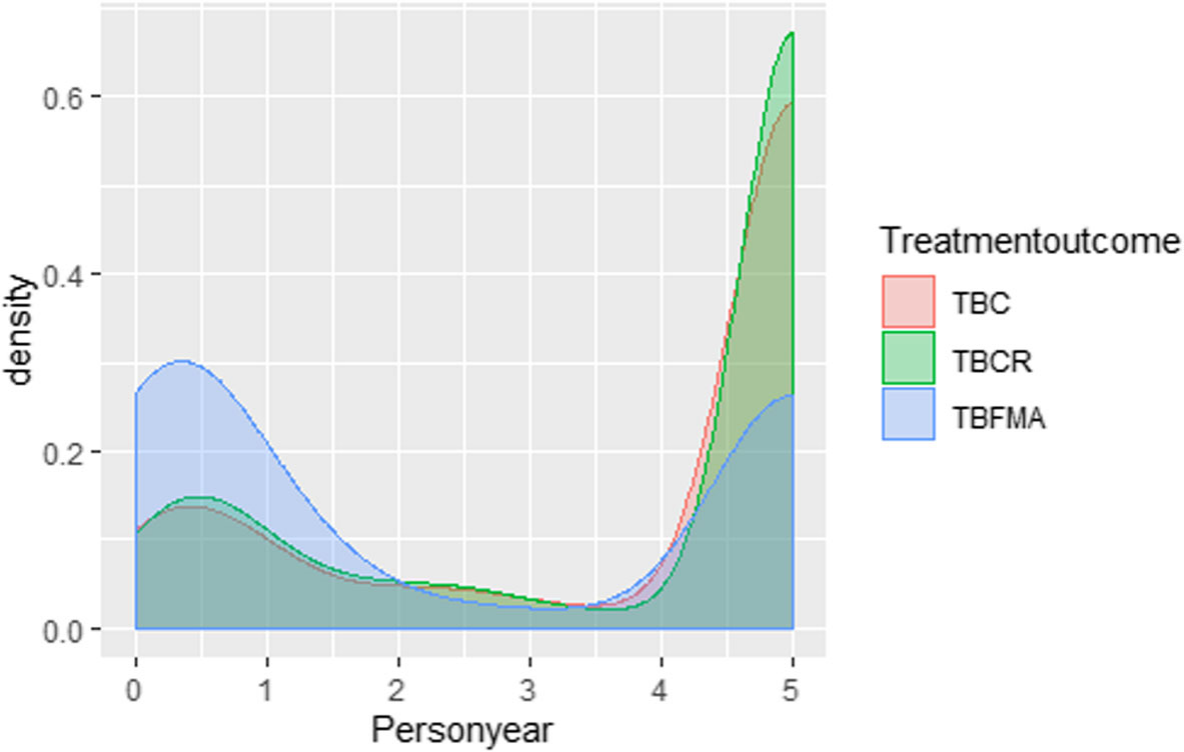
Density of mortality at the end of TB treatment (0.5 PYs), post-treatment year 1 (1 PYs), post-treatment year 2 (2 PYs), post-treatment year 3 (3 PYs), post-treatment year 4 (4 PYs), and post-treatment year 5 (5 PYs) among HIV/MTB coinfection patients treated with tuberculosis cure (TBC) (*n* = 680), tuberculosis complete regimen (TBCR) (*n* = 1289), and tuberculosis treatment failure, patients missing, adverse events (TBFMA) (*n* = 382) in Southern China

Mortality density analysis showed that the majority of mortality in the TBFMA group occurred during the first 6 months and mortality density was higher than those in TBC and TBCR group during the same period. On the contrary, the majority of mortality in both TBC and TBCR groups happened in the last year of follow-up (Fig. 3).

The HR value of mortality was calculated for TBC, TBCR, and TBFMA groups using Cox regression models. At the 60-month follow-up, the crude HR of mortality in the TBFMA group was 2.5 (95% CI: 2.1–3.1) times higher than that of the TBC group, and 2.6 (95% CI: 2.2–3.0) times higher than that of the TBCR group. A Wald chi-square test showed that the HR for mortality in the TBFMA group was significantly higher than those in the TBC and TBCR groups (*χ*^*2*^_*cure*_ = 95.4, *P* < 0.0001; *χ*^*2*^_*comp*_ = 131.8, *P* < 0.0001).

### HR adjusted by stratification variables

**Fig. 4:**
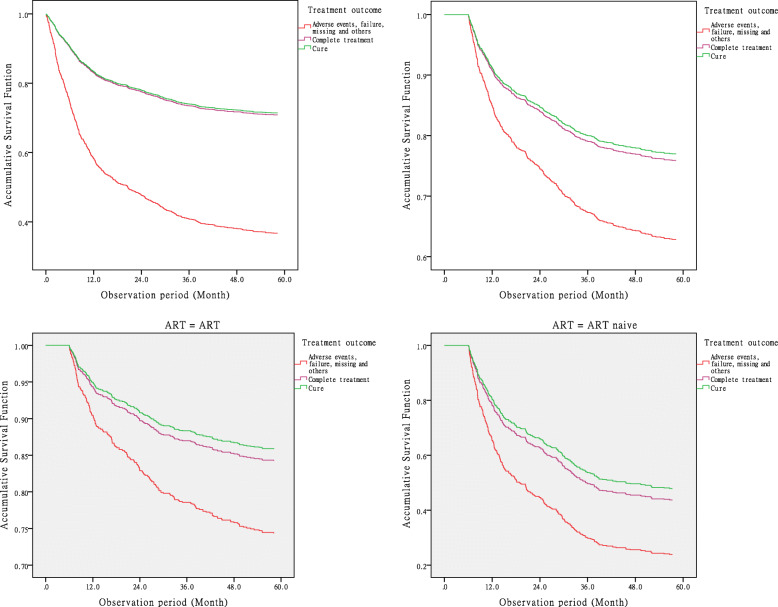
-1 and Fig. 4–2 Crude (4–1) and adjusted (4–2) accumulative Survival Function for mortality hazard risk at the end of TB treatment (6 months), post-treatment year 1 (12 M), post-treatment year 2 (24 M), post-treatment year 3 (36 M), post-treatment year 4 (48 M), and post-treatment year 5 (60 M) among HIV/MTB coinfection patients treated with tuberculosis cure (TBC) (*n* = 680), tuberculosis complete regimen (TBCR) (*n* = 1289), and tuberculosis treatment failure, patients missing, adverse events (TBFMA) (*n* = 382) in Southern China. Fig. 4–3 and Fig. 4–4 Adjusted accumulative Survival Function for mortality hazard risk at the end of TB treatment (6 months), post-treatment year 1 (12 M), post-treatment year 2 (24 M), post-treatment year 3 (36 M), post-treatment year 4 (48 M), and post-treatment year 5 (60 M) between stratifications of antiretroviral therapy (*n* = 362) and of antiretroviral therapy naïve (255) HIV/MTB coinfection patients treated with tuberculosis cure (TBC) (*n* = 680), tuberculosis complete regimen (TBCR) (*n* = 1289), and tuberculosis treatment failure, patients missing, adverse events (TBFMA) (*n* = 382) in Southern China

We adjusted the HR by deleting those who died within the initial 6 months of the study. The adjusted HR of mortality in the TBFMA group was 1.6 (95% CI: 1.2–2.0) times higher than the TBC group and 1.3 (95% CI: 1.0–1.7) times higher than the TBCR group, while the adjusted HR of the TBFMA group was significantly higher than the HR of TBC and TBCR groups (*χ*^*2*^_*cure*_ = 9.95, *P* = 0.0002; *χ*^*2*^_*comp*_ = 5.1, *P* = 0.024) (Fig. 4-1, 4–2).

Based upon preliminary analyses (see Table 1), ART, gender, age, occupation, and CD4 count were selected to compare the OR values in subgroups between TBFMA and the combination of TBC and TBCR. Results indicated that these selected factors were independent and unique factors that caused significant mortality across all groups except the group with CD4 count ≤200 μL (OR = 1.5, 95% CI:0.8–3.1, *χ*^*2*^ = 1.36, *P* = 0.24). Mantel-Haenszel stratified analysis results showed that TBFMA among males (OR = 3.9, 95% CI:3.0–5.1), those <50 years old (OR = 3.5, 95% CI:2.5–4.9), and with CD4 count >200 μL (OR = 3.4, 95% CI:2.6–4.6) were the top three hazard risks for death, compared to the HR of these subgroups in the control group (Table 3, Fig. 4–3, 4–4).

**Table 3:** Cox regression on Mortality risk of HIV/MTB co-infection in different stratification by TB treatment outcome

Stratification	TB Treatment outcome	β	SE	Wald	df	*P* value	Exp (β)	1-Exp (β) (%)	95.0% Exp(β) Confidential Interval	
									lower limit	Upper limit
ART and ART naïve	Adverse events, failure, missing			26.19	2.00	< 0.0001				
	Complete treatment	−.548	0.12	20.00	1.00	< 0.0001	0.58	42.18	0.45	0.74
	Cure	−.665	0.14	23.75	1.00	< 0.0001	0.51	48.55	0.39	0.67
Gender	Adverse events, failure, missing			21.38	2.00	< 0.0001				
	Complete treatment	−.521	0.12	18.16	1.00	< 0.0001	0.59	40.63	0.47	0.75
	Cure	−.575	0.14	17.83	1.00	< 0.0001	0.56	43.70	0.43	0.74
Age group	Adverse events, failure, missing			14.49	2.00	0.0007				
	Complete treatment	−.449	0.12	13.23	1.00	0.0003	0.64	36.17	0.50	0.81
	Cure	−.458	0.14	11.02	1.00	0.0009	0.63	36.75	0.48	0.83
CD4 group	Adverse events, failure, missing			17.87	2.00	0.0001				
	Complete treatment	−.486	0.13	14.35	1.00	0.0002	0.62	38.47	0.48	0.79
	Cure	−.567	0.14	16.07	1.00	0.0001	0.57	43.28	0.43	0.75

## Discussion

We found that the HIV prevalence among those first diagnosed with TB in Guangxi, China in 2011 was 5.6% with a cross-registration-system check and imputation method. This rate was higher than in previous studies based on surveys in hospitalization and routine TB surveillance [9, 12], There were 24,849 (58.8%) TB cases that had a documented HIV test in 2011 and the proportion of TB patients testing HIV-positive was 9.5% (2351/24,849), the percentage of those co-infected with HIV and TB in this study is consistent with testing results globally in 2017, but the proportion of TB patients testing HIV-positive in this study was less than the WHO estimate of 15% positive in 2017 [1].

Over 850 patients in this study died over the 5-year follow-up period. The survival of HIV/MTB patients may be impacted by the degree and timing of appropriate ART and anti-tubercular therapy. As previous studies have shown that ART improves the survival rate by at least 50% in individuals with HIV/MTB [13, 14], in 2012 the WHO endorsed a strategy recommending that ART should be initiated as early as possible for HIV-positive TB patients. Insufficient anti-tubercular therapy in HIV/MTB led to the inability to control mycobacterial infection and high mortality [15]. Our study found that inadequate TB therapy (TBFMA) was associated with a 2.97 (95% CI: 2.45–3.61) times higher mortality than those who were cured and a 2.91 (95% CI: 2.46–3.43) times higher mortality than for those who completed their TB regimen during the 60-month follow-up. Those HIV and TB coinfection patients with insufficient TB treatment were likely to die in an earlier stage of follow-up. Even after removing those who died during the 6-month duration of anti-tubercular therapy, a significant difference in mortality remained. After adjusting for those who were on ART, the OR was still 2.84 (95% CI: 1.99–4.05) times higher among those who TB treatment failed, missing, or experienced adverse events than in those who cured or completed TB treatment. This demonstrates that inadequate anti-tubercular therapy plays an important role in death for those with HIV/MTB coinfection. Similar results have observed in South Africa and Cote d’Ivoire [16, 17]. The reasons for high mortality in those whose TB treatment failed, missing, or experienced adverse events may due to MTB dissemination, overwhelming infections, or failure to achieve rapid immunological recovery [18, 19], but we did not have access to such clinical indicators in this study. Future research should focus on the relationship between immune recovery and treatment outcomes concerning HIV/MTB co-infection.

Risk factors for mortality among those HIV/MTB co-infected in China have addressed elsewhere [20, 21]. The majority of factors identified by hierarchical analysis in our study are consistent with the findings of previous studies. Adjusted by these risk factors, the HR for those whose treatment failed was still significantly higher than those who completed treatment and those who were cured. These results underline that uncontrolled TB with insufficient treatment should be considered as a leading cause of mortality among those with HIV/MTB coinfection. According to the WHO TB treatment guidelines [22], the standard treatment duration for cases of drug-susceptible TB is 6 months, and treatment for drug-resistant TB is longer. However, longer treatment duration may lead to low adherence to therapy and poor compliance has been associated with failure and drug-resistant TB [23–25], and lead to a high risk of medium to long term mortality. Methods to improve adherence such as shortening treatment duration, monitoring, and treatment of adverse events, and improving treatment success rates should be considered as a priority in clinical practice to reduce mortality risk of HIV/TB coinfection.

In our study, the median CD4 count found in the TBFMA group was l05 cells/μL (IQR: 25.5–268.5). This more advanced HIV/MTB stage leads to high mortality due to the effects of immune reconstitution inflammatory syndrome (IRIS). The enhanced immune function associated with ART initiation to treat HIV/MTB can lead to worsened clinical outcomes due to the increased severity of IRIS. TB-IRIS can categorize as “paradoxical” or “unmasking” in the mechanism. In paradoxical TB-IRIS, ART initiated in a patient with known TB, and clinical TB symptoms worsen after ART initiation. In unmasking TB-IRIS, patients with previously undiagnosed and untreated TB, such as latent TB, present with inflammatory features of TB after ART initiation [26]. Studies have shown that those with an advanced stage of HIV/MTB at the time of ART initiation, and the initiation of ART closer to the time of TB treatment initiated were consistent risk factors for TB IRIS [27, 28]; other research has found that the development of TB IRIS is associated with the rapid expansion of pathogen-specific CD4 cells following ART initiation [29, 30]. However, the results of other research are not consistent [31]. Our study cannot provide more details on TB IRIS because of the manner of data collection; thus, more research on pathophysiology and immunology are needed to define more precisely the mechanisms of TB-IRIS.

A strength of our study is that our results provide direct evidence for policymakers and stakeholders to highlight practical therapeutic developments and to review clinical outcomes of TB treatment for HIV/MTB co-infection. However, there were limitations to this study. First, we could not stratify data into pulmonary and extra-pulmonary TB to determine more precisely the impact of different TB types. Second, we could not analyze the role of TB IRIS on the mortality of HIV/MTB co-infection because of the method of data collection.

## Conclusions

HIV prevalence in TB registrations was 5.57% in Southern China; insufficient TB treatment increased long-term mortality nearly three times higher than those who were cured of TB and those who completed their TB regimen, the majority of the individuals who had inadequate TB treatment were likely to die in the 6 months of the follow-up period. The findings emphasize that sufficient TB treatment should be provided, the treatment success rates of TB treatment should be scaled up, and efforts toward early TB case-finding among people living with HIV should increase.

## Supplementary information

**Additional file 1 Supplemental Fig. 1**. Box plot with mean person year (white rhombus in charts), median person years (black line in charts) of followed-up among HIV/MTB coinfection patients treated with tuberculosis cure (TBC) (*n* = 680), tuberculosis complete regimen (TBCR) (*n* = 1289), and tuberculosis treatment failure, patients missing, adverse events (TBFMA) (*n* = 382) in Southern China. **Supplemental Fig. 2**. Mortality trend for percentage at the end of TB treatment (6 months), post-treatment year 1 (12 M), post-treatment year 2 (24 M), post-treatment year 3 (36 M), post-treatment year 4 (48 M), and post-treatment year 5 (60 M) among HIV/MTB coinfection patients treated with tuberculosis cure (TBC) (*n* = 680), tuberculosis complete regimen (TBCR) (*n* = 1289), and tuberculosis treatment failure, patients missing, adverse events (TBFMA) (*n* = 382) in Southern China.

## Data Availability

All data generated or analyzed during this study are included in this published article (and its supplementary information files).

## Abbreviations

IRQ: Interquartile range
IDU: Injection drugs users
ART: Antiretroviral therapy
M: Median
