# Supplementary material for: Insufficient tuberculosis treatment leads to earlier and higher mortality in individuals co-infected with HIV in southern China: a cohort study
Source: BMC Infect Dis. 2020 Nov 23;20:873. doi: 10.1186/s12879-020-05527-0 (PMC7682080; doi:10.1186/s12879-020-05527-0)
Supplement: Supplementary file 1 — Additional file 1 Supplemental Fig. 1. Box plot with mean person year (white rhombus in charts), median person years (black line in charts) of followed-up among HIV/MTB coinfection patients treated with tuberculosis cure (TBC) (n = 680), tuberculosis complete regimen (TBCR) (n = 1289), and tuberculosis treatment failure, patients missing, adverse events (TBFMA) (n = 382) in Southern China. Supplemental Fig. 2. Mortality trend for percentage at the end of TB treatment (6 months), post-treatment year 1 (12 M), post-treatment year 2 (24 M), post-treatment year 3 (36 M), post-treatment year 4 (48 M), and post-treatment year 5 (60 M) among HIV/MTB coinfection patients treated with tuberculosis cure (TBC) (n = 680), tuberculosis complete regimen (TBCR) (n = 1289), and tuberculosis treatment failure, patients missing, adverse events (TBFMA) (n = 382) in Southern China. [file 12879_2020_5527_MOESM1_ESM.docx]

Supplemental Figures


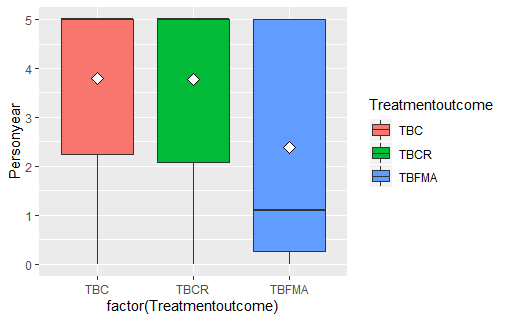


Supplemental Fig.1 Box plot with mean person year (white rhombus in charts), median person years (black line in charts) of followed-up among HIV/MTB coinfection patients treated with tuberculosis cure (TBC) (n=680), tuberculosis complete regimen (TBCR) (n=1289), and tuberculosis treatment failure, patients missing, adverse events (TBFMA) (n=382) in Southern China.


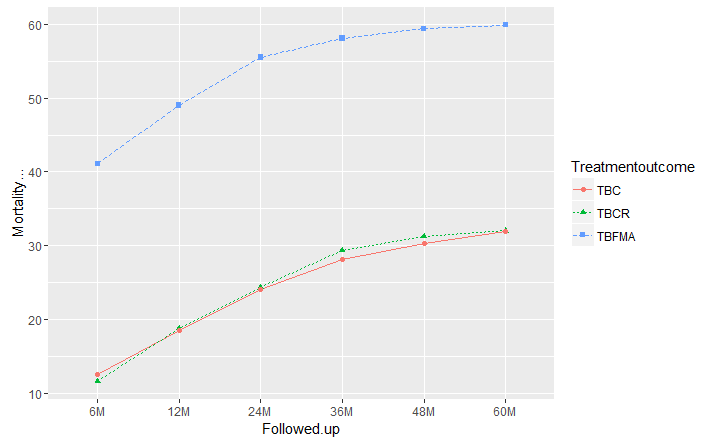


Month

(%)

Supplemental Fig.2 Mortality trend for percentage at the end of TB treatment (6 months), post-treatment year 1 (12M), post-treatment year 2 (24M), post-treatment year 3 (36M), post-treatment year 4 (48M), and post-treatment year 5 (60M) among HIV/MTB coinfection patients treated with tuberculosis cure (TBC) (n=680), tuberculosis complete regimen (TBCR) (n=1289), and tuberculosis treatment failure, patients missing, adverse events (TBFMA) (n=382) in Southern China.
